# Using Intervention Mapping to develop an intervention to promote healthy child weight development

**DOI:** 10.1093/eurpub/ckac130.239

**Published:** 2022-10-25

**Authors:** CT Bonnesen, RR Carlsson, LAS Brautsch, L Kierkegaard

**Affiliations:** National Institute of Public Health, Copenhagen, Denmark

## Abstract

**Background:**

Clear documentation of the understanding of the problem, development process, and content of interventions is essential to understand why interventions succeed or fail. Transparent reporting will enable future researchers to build on previous evidence and replicate or adapt interventions for new contexts. This paper describes the theory- and evidence-based systematic development of the Bloom Trial - a home-based intervention to promote healthy weight development among infants and children in Denmark.

**Methods:**

Development of the intervention is guided by the six-step planning tool the Intervention Mapping protocol. Step 1: Needs assessment including identification of risk factors in infancy and existing interventions, interviews with parents, and an organizational capacity assessment. Step 2: Development of program theory and matrices. Step 3: Selection of theoretical methods and practical applications for modifying personal and environmental determinants. Step 4: Development of intervention tools. Step 5: Planning of program adoption, implementation, and sustainability. Step 6: Generation of an evaluation plan.

**Results:**

The Bloom intervention is universal but with a strong focus on families with low socio-economic position and non-Danish ethnic background. It is aimed at first-time parents and addresses early risk factors for child overweight such as parental skills and healthy habits related to food and meals, movement, screen time and sleep, and introduce a new theme: sense of security in the family. It will be integrated in existing services delivered by community health nurses supplemented with extra elements such as telephone consultations, family groups and a video library.

**Conclusions:**

The transparency of the developmental process and theoretical, empirical, and contextual foundation of the Bloom Trial may enable future studies to build on our findings and accumulate knowledge to promote healthy weight development among infants and children.

**Key messages:**

- The development process resulted in a complex intervention addressing known risk factors for child overweight e.g., meals, movement, screentime and sleep, and introduce a new theme: sense of security.
- Transparency of the developmental process may enable future intervention studies to build on our findings and accumulate knowledge to promote healthy weight development among infants and children.

